# Should Alexa diagnose Alzheimer’s?: Legal and ethical issues with at-home consumer devices

**DOI:** 10.1016/j.xcrm.2022.100692

**Published:** 2022-07-25

**Authors:** David A. Simon, Barbara J. Evans, Carmel Shachar, I. Glenn Cohen

**Affiliations:** 1 Petrie-Flom Center for Health Law Policy, Biotechnology, and Bioethics, Harvard Law School, Cambridge, MA 02138, USA; 2 University of Florida Levin College of Law, Gainesville, FL 32611, USA; 3 University of Florida Herbert Wertheim College of Engineering, Gainesville, FL 32611, USA

## Abstract

Voice-based AI-powered digital assistants, such as Alexa, Siri, and Google Assistant, present an exciting opportunity to translate healthcare from the hospital to the home. But building a digital, medical panopticon can raise many legal and ethical challenges if not designed and implemented thoughtfully. This paper highlights the benefits and explores some of the challenges of using digital assistants to detect early signs of cognitive impairment, focusing on issues such as consent, bycatching, privacy, and regulatory oversight. By using a fictional but plausible near-future hypothetical, we demonstrate why an “ethics-by-design” approach is necessary for consumer-monitoring tools that may be used to identify health concerns for their users.

## Teaser

Voice-based AI-powered digital assistants, such as Alexa, Siri, and Google Assistant, present an exciting opportunity to translate healthcare from the hospital to the home. But building a digital, medical panopticon can raise many legal and ethical challenges if not designed and implemented thoughtfully. This paper highlights the benefits and explores the challenges of using digital assistants to detect early signs of cognitive impairment, focusing on issues such as consent, bycatching, privacy, and regulatory oversight. By using a fictional but plausible near-future hypothetical, we demonstrate why an “ethics-by-design” approach is necessary for consumer-monitoring tools that may be used to identify health concerns for their users.

## Main text

Voice-based artificial-intelligence (AI)-powered digital assistants are an exciting class of consumer-monitoring tools, of which Alexa, Siri, and Google Assistant are popular examples. As a whole, these products are beginning to form a web of “ambient intelligence,” a combination of AI and continuous monitoring, which enables ever-vigilant observation and analysis of human behavior in a designated physical space (“monitoring tools” or “tools”).^1^ This monitoring brings value to healthcare by, for example, bridging the doctor’s office with the patient’s home. But this medical panopticon also raises many legal and ethical issues.

In this paper, we examine some of these issues through the lens of a particular kind of monitoring technology (like Amazon’s Alexa), a particular disease class (Alzheimer’s and other degenerative brain diseases), and a particular country (the U.S.). We use a fictional, but plausible, near-future vignette—a digital assistant that has the capabilities to detect early signs of cognitive impairment using speech patterns detected through its monitoring tool—to illustrate the benefits and the concerns this kind of surveillance raises, including consent, information collection (bycatching) and its legal consequences, privacy, and federal regulation governing safety and effectiveness.

Although our vignette involves a particular voice-monitoring tool, the issues it raises and large parts of our analysis also apply to health-monitoring tools purchased by consumers writ large. In the same vein, our legal and regulatory analysis focuses on issues specific to the U.S., but similar legal and regulatory questions could arise in other countries, and many of the ethical issues raised by our analysis cut across borders.

### Hypothetical-use vignette

Amazon and other firms selling digital assistants currently offer services to monitor an elder person’s activity level, enable calls for help, and send alerts to designated relatives. Our vignette, set in 2030, envisions a world where natural language processing and AI software empower tools like Alexa and other digital assistants to monitor users for cognitive decline.^2^

Janice is a 77-year-old woman living alone. Her daughter, Maggie, works in another city and worries about Janice. Maggie buys Janice Amazon’s Hear, which is a new speaker with a built-in microphone and a voice-activated digital assistant. The Hear is equipped with a new AI tool that can detect early signs of cognitive impairment by identifying and analyzing patterns in speech and use of the monitoring tool. It even has the capability to produce a “speech report” that displays how speech patterns identified by the monitoring tool compare to the user’s past speech, as well as the speech of other users who own and use the tool. Janice and Maggie both felt the monitoring tool offered a promising way to help Janice remain in her beloved home while addressing Maggie’s concern about keeping her mother safe.

Over the next year, Maggie receives alerts that her mother’s speech patterns have changed and that she repeats questions. Maggie schedules a visit with her mother’s primary care physician and brings a printout of the speech report to the appointment. The physician refers Janice to a neurologist.

Concerned her mother could be a target for scammers, Maggie enrolls Janice in a service that monitors banking and credit card records for suspicious activity. She also hires a home care worker to assist Janice in her daily activities. Before long, Maggie receives reports of suspicious credit card activity. Janice cannot recall whether she made the purchases, and Maggie suspects Janice’s caretaker may have used Janice’s credit card. Janice’s neighbor lets Maggie know that Janice has bruises on her arms, which the caretaker states occurred while grabbing Janice to prevent a fall. Maggie is suspicious and reviews audio the monitoring tool captured, which reveals the caretaker yelling at Janice followed by loud banging noises.

### Why these new technologies hold promise

Continuous, in-home monitoring by AI-enabled tools offers several benefits. It can serve a notice or initiation function, alerting family members and perhaps even the patient herself to changes suggesting cognitive decline that might otherwise be missed by rapid, in-office assessment tools geriatricians routinely administer during clinical care encounters. In the vignette, Maggie became interested in the monitoring tool precisely because she could not be around to observe subtle changes to her mother’s mental state over time, changes that a physician might also miss. The monitoring tool detected changes that spurred Maggie to make an appointment with her mother’s physician.

Monitoring tools can also serve a confirmatory function for suspected cognitive decline. Family members might notice subtle changes over time but sometimes feel reluctant to raise the issue either with the patient or their physician. Patients may actively hide their difficulties from loved ones and physicians out of embarrassment or fear of losing independence. Objective continuous monitoring can provide confirmation of patient struggles, helping family members to initiate treatment or to speak out during a consultation. In an ideal future world, alerts could be directed to a trusted physician or geriatric care provider, although this presumes the nation’s already-strained geriatric care workforce would have capacity to monitor and act on a new stream of incoming alerts.

By detecting unsafe use of credit cards, smartphones, internet services, or other home appliances (such as leaving a stove on), monitoring tools can even be autonomy enhancing for the user being monitored. As cognitive and mobility impairments curtail a person’s ability to venture out into the world, online shopping and smartphones offer crucial lifelines to necessities like grocery deliveries and to social interaction. In other words, monitoring tools offer “guard rails” that enable users experiencing cognitive decline to enjoy continued access to pleasures, conveniences, and necessities that otherwise might pose unacceptable risks of harm to themselves or to others.

### Interactions with the intended and incidental users

Despite these benefits, monitoring technologies pose concerns, which can include consent, bycatching, and other legal consequences.

#### Consent

The vignette described above is somewhat vague regarding Janice’s consent to bringing a monitoring tool into her home, activating its speech report function, and determining with whom its analysis will be shared. But consent is a cornerstone of both U.S. law and medical treatment. It requires “informed exercise of a choice, and that entails an opportunity to evaluate knowledgeably the options available and the risks attendant upon each.”^3^

The vignette raises complicated questions about whether Janice’s consent met this standard. If a physician evaluated her mental state in a clinic, the setting and interaction itself would give Janice notice she is being monitored. Janice would also have the opportunity to raise concerns about the test or refuse it outright. The vignette offers no such professionalized encounter. It involves an ordinary consumer purchasing a monitoring tool that could generate useful information for the healthcare system but might not have undergone FDA review for use as a diagnostic device. Rather than an interactive informed consent process with a physician, Janice is more likely to have been offered a click-through user agreement: a long, dense legal document that explains how the company could use data the monitoring tool generates. What is more, most users never even read such agreements; one memorable example is a Wi-Fi company that signed up 22,000 people for free public Wi-Fi who had inadvertently agreed to 1,000 hours of community service, including toilet cleaning and “relieving sewer blockages.” (https://www.npr.org/2019/03/08/701417140/when-not-reading-the-fine-print-can-cost-your-soul). In the unlikely event that Janice was attuned to the detailed terms of the original agreement, future software updates may change the nature of what information is collected and how it is stored and analyzed.

The user agreement is also almost certainly a contract of adhesion, a take-it-or-leave-it proposition without an opportunity to request changes. The developer of the monitoring tool might automatically and non-negotiably “bundle” the various features: Janice may want her digital assistant to play music or tell her the weather but object to its recording her or monitoring her cognitive state or not want the provider to store data that could enable reidentification. Unfortunately, the firm offering the tool, not Janice, likely decides how to use the information the monitoring tool collects, a decision that it can change merely by alerting Janice that the “terms of service” have changed.

All of this would be a challenge to meaningful consent for any person, but the issues become trickier for someone experiencing cognitive decline. Even if Janice fully understood what the monitoring tool would record and how it would be used when it was *first* powered on in her home, she might face periods where she forgets it is listening or with whom it shares information. The use could change with the terms of service, and Janice might not have the capacity to consent when they do, raising something of a paradox: a valid consent must precede cognitive decline, but cognitive decline may have commenced before consent was obtained, which raises questions about the appropriateness of both the model of consent and how it would be implemented for monitoring tools that detect cognitive decline.

#### Bycatching

Apart from concerns about Janice’s data, home-monitoring tools also may capture others' data. The Cambridge Dictionary defines a bycatch as “fish or other sea creatures that are caught unintentionally by people who are trying to catch other types of fish.” (https://dictionary.cambridge.org/us/dictionary/english/bycatch). It is also a useful term for the way a monitoring tool collecting data on one individual might collect data on other individuals in the same environment. Here, we are primarily focused on instances where bycatching is incidental rather than an intended purpose of the tool.

In this vignette, the tool captures the speech of the caretaker yelling at Janice, which is a sort of “incidental finding” that stems from the recording itself. But one could also imagine instances where bycatching results from the tool processing information about cognitive decline, but for the wrong person, such as a roommate. If the cognitive decline it detects is not Janice’s decline but her roommate’s (or even the caretaker’s), the monitoring tool might incorrectly activate alerts for *Janice*, potentially disabling her bank transfers. Meanwhile, Janice, and not her roommate, could have access to her roommate’s medical information, such as the speech reports, produced by the tool, all without the roommate’s consent. These and other scenarios—such as capturing the caretaker's phone conversations with lawyers or financial advisors, or even flirtatious exchanges with a potential suitor—raise challenging ethical and legal questions.

#### Legal consequences

Nevertheless, we may feel positive about the bycatching in the vignette because potential elder abuse was detected and could be remedied. However, legal and ethical questions remain. Consider, for example, whether a firm, such as Amazon, providing a digital assistant service ought to be obligated to report such abuse to the police or adult protection services. Fourteen U.S. states have adult protection statutes that mandate reporting to state agencies by “any person” who becomes aware that an elderly person is suffering neglect.^4^ A service or software provider that develops data suggesting a user is cognitively impaired and not receiving adequate care might, in some states, have a legal duty to report this information, potentially triggering official wellness inquiries or even guardianship proceedings. What triggers this obligation is not an easy question. Consider the recording in the vignette: although it suggests caretaker abuse, it is also not inconsistent with the caretaker’s story.

While suspected abuse may trigger reporting obligations that could have legal consequences for the abuser, suspected cognitive impairment can have profound legal consequences for the person using monitoring tools. For example, inferences the tool draws about a user’s cognitive status—whether accurate or not—might influence decisions to seek guardianship for an elderly person. Guardianship limits the right to make one’s own medical decisions, to vote, to manage property, and to decide where to live. Because guardianship proceedings are civil, rather than criminal, a person’s statements made to a digital assistant could be used as evidence of cognitive impairment. When the need for guardianship is borderline or based on inaccurate data, the costs and hurdles of reclaiming one’s full autonomy are high.

Users may have the perception that because these tools were not “officially” prescribed by physicians or used in the clinic that their use will not have serious legal or medical consequences. This could lead to an inappropriately lackadaisical “why not” approach to incorporating these tools into the home.

### The limitations of existing regulatory regimes

Beyond issues of consent, bycatching, and legal consequences, Janice's and Maggie’s use of the monitoring tool in our vignette illustrate how our pre-existing regulatory regimes for privacy and safety/efficacy struggle to categorize and regulate the use of monitoring tools to detect cognitive decline.

#### Privacy

The Health Insurance Portability and Accountability Act (HIPAA), 42 U.S.C. § 1320 et seq., exemplifies the regulatory gaps surrounding the use of home-monitoring tools to detect cognitive decline. HIPAA, together with its federal regulations, 45 C.F.R. §§ 160, 162, and 164, ensure the privacy and security of protected health information (PHI)—as well as access to that information—but only if the PHI is generated or stored by (or transmitted to) a “covered entity,” the definition of which does not typically include firms, like Amazon, that sell digital assistants. HIPAA, with its primary focus on traditional healthcare actors and medical records, does not reach the myriad ways we now generate and record health information.^5^

This means that when the monitoring tool records information about Janice’s cognitive capabilities, data privacy protections may be quite limited despite information about cognitive decline “feeling” very medical. Without a proper understanding of data privacy law, both women could lose the opportunity to make privacy choices for Janice. It also means that firms can use and sell user data. Some of these uses may be beneficial (e.g., potentially lowering the cost of the monitoring tool or locking her credit card to prevent fraudulent activity), while others may be more suspect (e.g., targeting Janice with ads for dubious “memory aid” supplements). Even more robust protections that would protect Janice’s use of monitoring tools—like those offered for “data concerning health” by the European Union’s General Data Protection Regulations (GDPR) and state privacy laws—would not apply to Janice unless the firm meets specific requirements.^6^^,^^7^

Merely extending HIPAA to cover at-home health-monitoring tools would not, however, solve Janice and Maggie’s data privacy problems. Congress designed HIPAA for clinical healthcare where many users of data (e.g., physicians) are already bound by other state and federal laws requiring them to act as “informational fiduciaries” of the data they handle, and HIPAA lacks strong confidentiality requirements of its own.^8^^,^^9^

#### FDA regulation

In some cases, existing Food and Drug Administration (FDA) regulation of monitoring tools used to identify cognitive changes may not adequately ensure that the product is safe and effective for the use to which it is put. FDA regulates medical “devices,” which are “*intended for use* in the diagnosis of disease or other conditions ... .” Monitoring service providers may market their monitoring tools as “low-risk general wellness products,” otherwise defined as products that “(1) are intended for only general wellness use…, and (2) present a low risk to the safety of users and other persons.”^10^ As examples, FDA cites fitness trackers that “allow awareness of one’s exercise activities to improve or maintain good cardiovascular health” and diet trackers that “manage dietary activity for weight management and alert the user, healthcare provider, or family member of unhealthy dietary activity.” Service providers could position their cognitive assessment tools as merely tracking speech patterns to allow awareness of mental acuity.

Because general wellness products are not evaluated by FDA for safety or effectiveness, Maggie and Janice would not have any “reasonable assurance” that the home-monitoring tool safely and effectively identifies concerning changes in speech patterns if the monitoring tool is a low-risk general wellness product.^11^ Thus, they would not have reasonable assurance that the tool reliably identifies cognitive decline, which could cause Maggie or Janice unnecessary distress or confound a physician who struggles to understand the monitoring tool’s evidentiary value. While FDA’s policy on low-risk general wellness products reflects a sound approach to evaluation based on their level of risk, regulators should be sensitive to potential “crossover” uses by consumers when companies “skate the line” between unregulated wellness products and regulated medical devices.^12^

Even if a consumer-grade monitoring tool is a device reviewed by FDA rather than an unreviewed general wellness product, consumers might use it outside of its intended use, particularly if it is available without a prescription. In the vignette, for example, the tool could be a device intended for use in diagnosing mild cognitive impairment in individuals aged 80 to 100 under conditions of constant use in a one- or two-person household. However, if Janice lived with her extended family or turned off her device periodically, it may not function properly. Likewise, if Maggie attempted to use the device to diagnose her younger, 45-year-old sister with Alzheimer’s, the device’s analysis may be inaccurate because the sister is outside the intended age range of the device and because the device is not intended for that purpose.

Relatedly, the monitoring tool may be too unreliable to serve as a basis for life-altering decisions. For example, the natural language processing software and AI tools used to produce the “speech report” in the vignette might have been trained on data (partly or wholly) from patients aged 60 to 70, rendering inferences biased and unreliable for assessing the cognitive status of older populations. Factors affecting elderly populations, such as hearing impairment, might alter a user’s interactions with the monitoring tool, leading to erroneous signals that the user is confused when, in fact, they simply cannot hear the digital assistant’s synthesized voice.^13^

### Conclusion

It is tempting to call for expansion of existing regulatory frameworks to address the concerns described above, but it is not clear that traditional, top-down regulatory approaches can be effective for the flood of AI home health-monitoring tools arriving over the next 5–10 years. Agencies like the FDA, already stretched thin by current oversight responsibilities, may lack staffing, capacity, and legal authority to offer comprehensive oversight of these tools. Even if they had the capacity, 20th-century regulations presume product-development lifecycles and industry structures that might not be a good fit for the fast-evolving ecosystem of start-ups and software developers that would need to be regulated.

As for privacy, simply subjecting monitoring service providers to traditional privacy laws, which stress consent and de-identification, may not protect consumers’ privacy in a world where consumers embrace exciting AI tools without thoughtful, truly informed consent, and where de-identification is ever easier to thwart. These issues are even more pronounced when the populations monitored include individuals with declining mental acuity, such as potential Alzheimer’s patients.

We therefore call on policymakers to widen the type of solutions under consideration, recognizing that technical solutions may be more powerful than hard-to-enforce legal solutions. In particular, there is a role for approaches that integrate privacy and ethical standards into the design of AI monitoring tools.^14^ Firms designing monitoring tools should be required to incorporate features promoting transparency, fairness, human agency, data protection, accountability, oversight, and well-being.^15^ An example would be a visible indicator that reminds users (and bystanders) when monitoring is taking place, with regular queries to allow the user to turn off the monitoring tool and reminders of how the data might be used. It might also mean equipping digital monitoring tools with the capacity to explain all the functionalities available, mimicking more closely the explanations that precede medical decision making during physician office visits. Such ethics-by-design and privacy-by-design approaches might also require unbundling various functions of these monitoring tools and enabling more choice by the consumer. Moving forward, it is important to recognize that monitoring tools like the one discussed in our vignette are neither simply extensions of the physician’s office into the home nor simple consumer products. Rather, they occupy a middle ground that requires subtle thinking about how they can ethically benefit patients very much in need of the help.
